# Dynamic Responses and Crack Propagation of Rock with Crossed Viscoelastic Joints Under Blasting Loads

**DOI:** 10.3390/ma18030548

**Published:** 2025-01-25

**Authors:** Chengyang Li, Dongju Jiang, Jinhai Zhao, Tuo Zhang, Renfei Kuang

**Affiliations:** College of Mechanics and Engineering Sciences, Hohai University, Nanjing 211100, China; lichengyang_1214@163.com (C.L.); zhangtuo9922@163.com (T.Z.); kuangrenfei1104@163.com (R.K.)

**Keywords:** viscoelastic joints, stress wave propagation, crack propagation, blasting

## Abstract

To investigate the propagation of stress waves in viscoelastic joints under blasting loads, and their impact on crack propagation and dynamic response in rock masses, a numerical model incorporating intersecting viscoelastic joints was developed using LS-DYNA. This study focuses on the influence of various joint geometric parameters, including thickness and angle, on stress wave propagation and damage patterns in rock. The Riedel–Hiermaier–Thoma (RHT) model was employed to simulate the dynamic behavior of rock, while the Poynting–Thomson model was used to describe the viscoelastic properties of the joint fillings. The simulation results provide detailed insights into the principal stress, displacement, and particle vibration velocity around the joints. Based on the stress wave propagation theory, the velocity transmission coefficients were calculated to quantify the attenuation of stress waves across the joints. The findings demonstrate that viscoelastic joint properties significantly affect the damage patterns in the rock mass. Specifically, the area of the crushed zone and the width of cracks on the blasting side are proportional to joint thickness, while crack propagation at the joint tips is governed by differences in principal stress. Moreover, the propagation of vibration velocity is notably weakened at the second joint, highlighting the critical role played by joint characteristics in stress wave dynamics. These results underscore the complex interaction between joint properties and stress wave behavior in rock masses, providing valuable insights for optimizing blasting designs and improving the safety of underground engineering projects.

## 1. Introduction

Joints and fractures, which are widely present in natural environments, divide the rock mass into irregular and discontinuous geological bodies. And due to the filling of soft soil and groundwater, the joints often possess viscoelastic properties. This also leads to changes in the strength and deformability of the rock. During blasting operations, when the stress waves generated by the blast propagate to these interfaces, phenomena such as reflection and transmission will occur. This induces irregular crack propagation, which may result in unexpected blasting outcomes. This can even lead to the instability of the surrounding rock, severely impacting construction safety and efficiency. Therefore, exploring crack and wave dynamics in rock masses with viscoelastic joints is vital for optimizing blasting performance.

In recent decades, researchers have conducted numerous theoretical analyses on the issue of stress wave propagation in rock masses containing joints [1,2,3]. Li and Ma [4] proposed a time-domain recursive method (TDRM) to quantitatively analyze the interaction between obliquely incident P wave or S wave and linearly elastic rock mass joints and derived wave propagation equations. This method was further extended to address the problem of stress wave propagation in rock containing multiple sets of parallel joints [5] and intersecting joints [6,7]. Li [8] and Zhu et al. [9] used the virtual wave source method to account for the viscoelastic behavior of joint fillings, obtaining analytical solutions for waves passing through jointed rock masses at arbitrary angles. Wang et al. [10] extended this method by introducing different equivalent viscoelastic models and deriving the stress wave propagation equations. From an experimental analysis perspective, scholars tend to observe the changes in stress and particle vibration velocity on both sides of the joint to study the interaction between stress waves and joints. This allows them to determine the dynamics of stress wave propagation under different joint geometric parameters. For example, based on experimental data, Zhu et al. [11] derived the analytical solution for wave propagation through a single joint by curve fitting. Liu et al. [12] conducted SHPB impact tests on rock masses with intersecting joints, analyzing the dynamic mechanical properties, energy dissipation, and failure patterns of intersecting jointed rock masses. Chang et al. [13] studied the anisotropic strength characteristics of intersecting joint specimens at different angles through uniaxial compression tests on simulated rock-like materials. Wu et al. [14] studied the fracture process of rock samples under uniaxial compression. The results showed that the strength varied in a “V” shape with the joint angle, and the main crack was the dominant factor in crack propagation.

As an effective and precise tool, numerical simulations are commonly utilized to investigate the impact of joints on crack patterns and stress wave behavior under explosive loading. For example, Zhou et al. [15] studied the blasting process in jointed rock masses and established the relationship between the transmission and reflection properties of the rock mass and the characteristics of the joints. Ding et al. [16] used LS-PREPOST to simulate the mode conversion process of stress waves at the interfaces of layered composite rocks and explored the crack development patterns. Zhu et al. [17] used the discrete element method to examine how wave propagation is influenced by intersecting joints in rock masses. A comparison with analytical solutions and experimental data showed that joint mechanical and geometrical parameters determine wave propagation. Jayasinghe et al. [18] simulated the blasting process by constructing an SPH-FEM coupled model to study the impact of discontinuities on the evolution of blast-induced damage. Similarly, Wang and Konietzky [19] combined FEM and DEM to study the crack propagation patterns in two typical jointed rock masses and discussed the impact of in situ stress and free surfaces on crack evolution.

To further study the mechanism of crack evolution and conduct a quantitative analysis and assessment, Shang et al. [20,21,22] proposed that the failure mode of rock masses is generally controlled by structural planes, and the mechanical properties of these structural planes are influenced by the distribution and geometry of intact rock bridges. Zou et al. [23,24], through experiments on gypsum specimens under dynamic and quasi-static loading, discovered that the crack propagation mode is influenced by the strain rate. Under quasi-static compression conditions, the failure mode is dominated by tensile wing cracks, whereas under dynamic compression conditions, it is dominated by shear cracks. Based on the distribution of principal stress and displacement around the joint surface, Jiang et al. [25] derived the dynamic stress intensity factor (DSIF) at the joint tips and analyzed the crack propagation mechanism of the joint surface. Yilmaz and Unlu [26] discovered that under blasting loads, rock’s tensile failure zone increases with the differential principal stress. Therefore, the principal stress difference can be considered a primary criterion for evaluating the direction of crack development. Liu et al. [27] proposed a new method based on digital image correlation (DIC) analysis and suggested that variations in shear stress distribution near defects could be the main reason for changes in crack initiation mechanisms at different inclinations.

**Figure 1:**
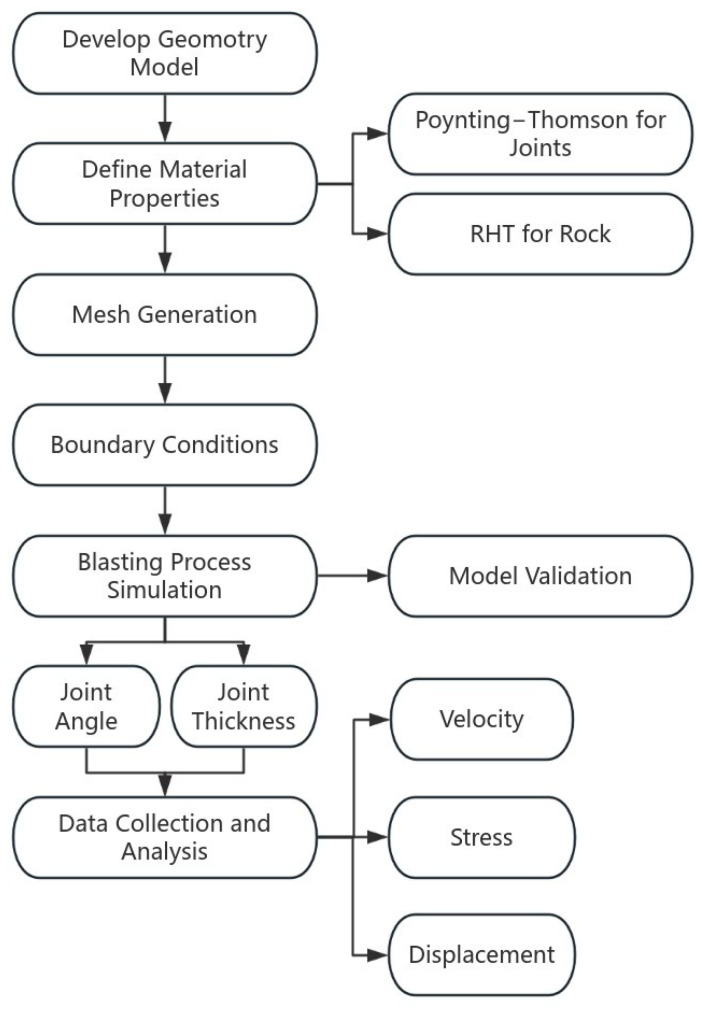
Methodology flowchart.

Although research on stress wave propagation has a long history, most studies have focused on single joint or parallel joints. The distribution of intersecting viscoelastic joints is more varied. Therefore, accurately assessing the impact of multiple viscoelastic joints on blasting effects in rock masses is crucial for ensuring the safety of underground engineering projects. This paper utilizes ANSYS/LS-DYNA R11.0 to conduct simulations of the blasting process in rock masses containing viscoelastic joints. The effect of joint geometries (thickness, angle) on rock mechanics response and blasting effects was analyzed, which provides certain guidance for rock blasting engineering. A flowchart of the methodology adopted is shown in Figure 1.

## 2. Fundamental Principles of Blasting Simulation

### 2.1. RHT Model

Given the similarity in strength and elasticity between concrete and natural rock, it is possible to use the constitutive model of concrete to simulate the behavior of rock subjected to dynamic loading. Among them, the Riedel–Hiermaier–Thoma (RHT) model and Holomquis–Johnson–Cook (HJC) model have been commonly employed for numerical simulations of rock blasting, impact, and penetration problems. Wang et al. [28] found that both models can describe the formation of crushed zone and cracks in blasting problems. However, the RHT model provides a more comprehensive description of rock behavior under blasting loads, including strain hardening, damage accumulation, and pressure-dependent strength, making it more suitable for our study [29]. Although the RHT model does not explicitly account for viscoelasticity, we combined it with the Poynting–Thomson model to accurately describe the viscoelastic behavior of joint fillings in this study. This hybrid approach allows us to effectively capture both the dynamic fracture of rock and the viscoelastic response of joints.

The RHT model under loading progresses through three sequential stages: the elastic phase, the linear phase, and the damage softening phase, where the equivalent stress value on the failure surface can be defined as follows [29]:

(1) $$\sigma_{f}(p,\theta ,\dot{\varepsilon })=f_{c}\sigma_{f}^{*}(p_{s})R_{3}(\theta )F_{r}(\dot{\varepsilon })$$

where θ is the Lode angle; $\sigma_{f}^{*}(p_{s})$ is the equivalent stress intensity of the quasi-static failure surface compression meridian; $F_{r}(\dot{\varepsilon })$ is the strain rate dynamic enhancement factor; and quasi-static pressure can be expressed as $p_{s}=p/F_{r}(\dot{\varepsilon })$. The Lode angle factor $R_{3}(\theta )$ is proposed to characterize the reduction in the failure strength of the failure surface compression meridian. The strain rate enhancement factor $F_{r}(\dot{\varepsilon })$ under different loads of tension and compression can be derived by the following equation [29]:

(2) $$F_{r}(\dot{\varepsilon })=\left\{\begin{array}{cc} {\left(\frac{\dot{\varepsilon }}{{\dot{\varepsilon }}_{0}^{c}}\right)}^{\beta_{c}} & p\geq f_{c}/3\\ \frac{p+f_{t}/3}{f_{c}/3+f_{t}/3}{\left(\frac{\dot{\varepsilon }}{{\dot{\varepsilon }}_{0}^{c}}\right)}^{\beta_{c}}+\frac{p-f_{c}/3}{-f_{t}/3-f_{c}/3}{\left(\frac{\dot{\varepsilon }}{{\dot{\varepsilon }}_{0}^{c}}\right)}^{\beta_{t}} & -f_{t}/3<p<f_{c}/3\\ {\left(\frac{\dot{\varepsilon }}{{\dot{\varepsilon }}_{0}^{t}}\right)}^{\beta_{t}} & p\leq -f_{t}/3 \end{array}\right.$$

where $f_{t}$ is uniaxial tensile strength; $\beta_{c}$ is the compressive strain rate exponent; $\beta_{t}$ is the tensile strain rate exponent; and $f_{c}$ is uniaxial compressive strength.

The equivalent stress on the initial elastic limit surface of the material is derived from the equivalent stress on the failure surface combined with the cap function and elastic strength parameters [29]:

(3) $$\sigma_{el}(p,\theta ,\dot{\varepsilon })=f_{c}\sigma_{f}^{*}(p_{s,el})R_{3}(\theta )F_{r}(\dot{\varepsilon })F_{el}(p^{*})F_{c}(p^{*})$$

Damage begins to accumulate when the stress exceeds the critical failure strength $\sigma_{el}$. As a result, the material transitions into the damage-softening phase. At this stage, the damage variable D can be expressed as the ratio of the cumulative equivalent plastic strain increment to the final failure equivalent plastic strain [29]:

(4) $$D=\sum \frac{\Delta \varepsilon_{p}}{\varepsilon_{p}^{f}},\varepsilon_{p}^{f}=D_{1}{\left(p^{*}-HTL^{*}\right)}^{D_{2}}\geq \varepsilon_{p}^{m}$$

where $\Delta \varepsilon_{p}$ is the equivalent plastic strain increment; $D_{1}$ and $D_{2}$ are the material parameters; and $\varepsilon_{p}^{m}$ is the minimum equivalent plastic strain at material failure.

### 2.2. Blasting Load Analysis

Various equations of state have been proposed by researchers to characterize the shockwaves generated by explosions. However, only a limited number of these models are capable of accurately describing and predicting the behavior of high explosives. Among them, the Jones–Wilkins–Lee (JWL) equation, as a highly comprehensive equation of state, is widely used in single-hole explosion simulations. The pressure P generated by high explosives can be defined by the JWL equation [30], as shown in Equation (5).

(5) $$P=A\left(1-\frac{\omega }{VR_{1}}\right)e^{-R_{1}V}+B\left(1-\frac{\omega }{VR_{2}}\right)e^{-R_{2}V}+\frac{\omega e}{V}$$

where A, B, ω, $R_{1}$, $R_{2}$ are explosive parameters; V is the relative volume $v_{1}/v_{0}$; and e is the internal energy parameter.

## 3. Establishment of Numerical Model

### 3.1. Model Parameters and Verification

To calibrate the RHT model and validate the simulation results in this study, laboratory-scale blast test data from Banadaki [30] were used. These tests were conducted on two distinct rock types: Laurentian granite and Barre granite. For the purpose of comparison, only the results based on Barre granite are considered in this analysis. Banadaki [30] conducted a comprehensive sensitivity analysis of the RHT model parameters and validated the numerical stability through mesh convergence studies and experimental comparisons. The sample in these tests had a diameter of 144 mm, with a centrally located borehole of 6.45 mm in diameter.

The explosive employed in the tests was a No. 2 emulsion explosive, a commonly used material in tunnel blasting. The material properties and corresponding equation of state for the explosive are provided in Table 1. Model parameters of the explosives: D is the detonation velocity, P_CJ_ is the Chapman–Jouguet pressure, and E_0_ is the detonation energy per unit volume. The procedure for determining the RHT model parameters is outlined in Xie et al. [29], and the specific parameters for Barre granite are listed in Table 2. For the RHT model, G is the elastic shear modulus, f_c_ is the compressive strength, and P_el_ and P_co_ are the crush and compaction pressure, respectively. Detailed material properties for the air and viscoelastic-filled joints are presented in Table 3 and Table 4. For air, C4 and C5 are constants, and E_0_ represents initial internal energy. For the joint fillings, E is the elastic bulk modulus, G_0_ and G_L_ are the short-time and long-time shear modulus, respectively, and β is the decay constant.

**Figure 2:**
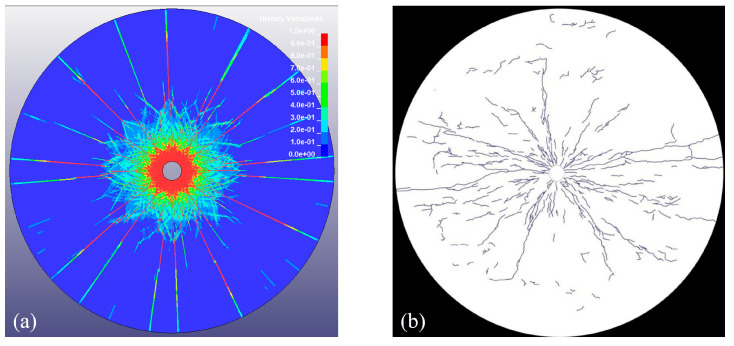
Crack patterns of Barre granite sample under blasting loads. (**a**) Numerical simulation; (**b**) blast experiment [30].

Figure 2 compares the blast-induced crack patterns observed in the experiment [30] with those obtained from the numerical model in this study. In the simulation, cracks are represented by damage contour lines that range from 0 (indicating no damage, shown in blue) to 1 (representing complete damage, shown in red). Intermediate colors between these extremes correspond to varying degrees of damage within the rock. It can be seen from Figure 2 that the results indicate a good agreement between the computational results and the experimental results. The reasonable computational results of the numerical model in this study provide a foundation for the subsequent research.

**Figure 3:**
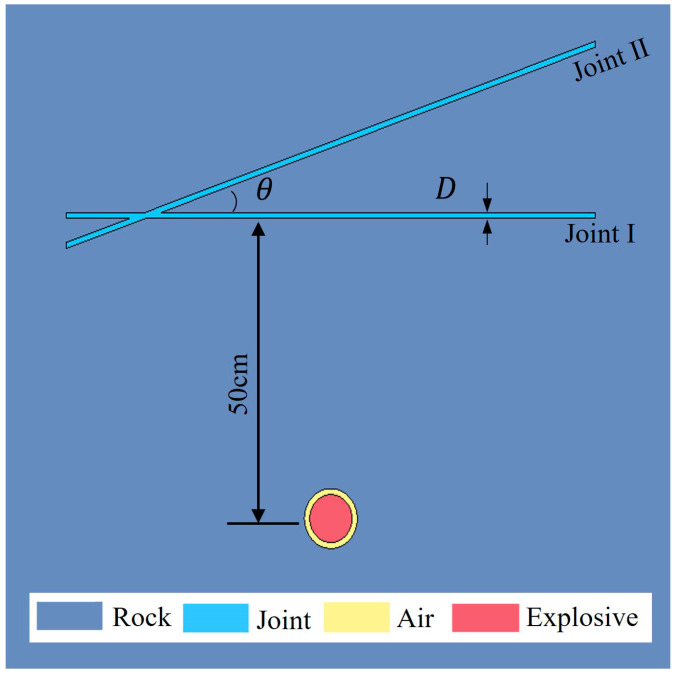
Schematic diagrams of the charge and joints in the models.

To study the impact of intersecting joints on stress wave propagation in rock masses, this paper constructs a model of a rock mass with intersecting joints, as shown in Figure 3. The model elements are set as solid elements. The rock and joint filling models are defined using the Lagrangian algorithm, while the air and explosives are defined using the arbitrary Lagrange–Euler (ALE) coupling algorithm. To address the issue of mesh distortion caused by large deformations during the blast simulation, the contact between the rock, joints, explosives, and air is set using the fluid–solid coupling algorithm. It can be handled by using the *CONSTRAINED_LAGRANGE_IN_SOLID option in LS-DYNA.

### 3.2. Geometric Model and Mesh Division

The dimensions of the rock model are 300 cm × 300 cm × 0.5 cm. The model employs a single-hole blasting configuration with decoupled charges, where the borehole diameter is 5 cm and the charge diameter is 4 cm. The borehole is positioned at the center of the model, with the detonation point located at the center of the borehole. To reduce wave distortion, the maximum element size is typically controlled within 1/10 of the wavelength λ. Therefore, the mesh consists of hexahedral elements with a side length of 0.5 cm. Since rock stress wave propagation is typically considered a plane strain problem, the model is simplified to a quasi-3D model with a thickness of one element layer. Joint I is in the horizontal direction, located 50 cm from the borehole center, with a length of 100 cm. Joint II has a horizontal inclination angle of θ. Figure 3 shows the condition for two joints with a thickness of 1.0 cm and an angle of 20°.

To simulate the actual infinite boundary conditions, the boundaries around the X-Y plane of the rock mass model are set as non-reflective boundary conditions. The displacement in the *z*-axis direction is constrained along the entire boundary of the model to ensure that the model remains in a plane strain state throughout the entire blasting process.

## 4. Numerical Results

### 4.1. The Effect of Joint Angle θ on Stress Wave Propagation

The propagation process of detonation waves is usually represented as damage zones in the surrounding rock mass [31,32]. After the explosive is detonated, a blast shock wave will strongly impact the rock around the borehole. This leads to large displacements of rock particles, forming a crushed zone. A blast wave consumes most of the explosive energy on the rock surrounding the borehole, so the energy of the wave rapidly decreases and gradually transforms into vibrational stress waves. During propagation, the vibrational stress waves generate tensile stress components. Since the tensile strength of the rock is much lower than its compressive strength, this tensile stress component causes radial cracking in the rock mass. When the stress waves encounter joints, the crack propagation in the rock mass becomes more complex due to the transmission and reflection of the stress waves.

**Figure 4:**
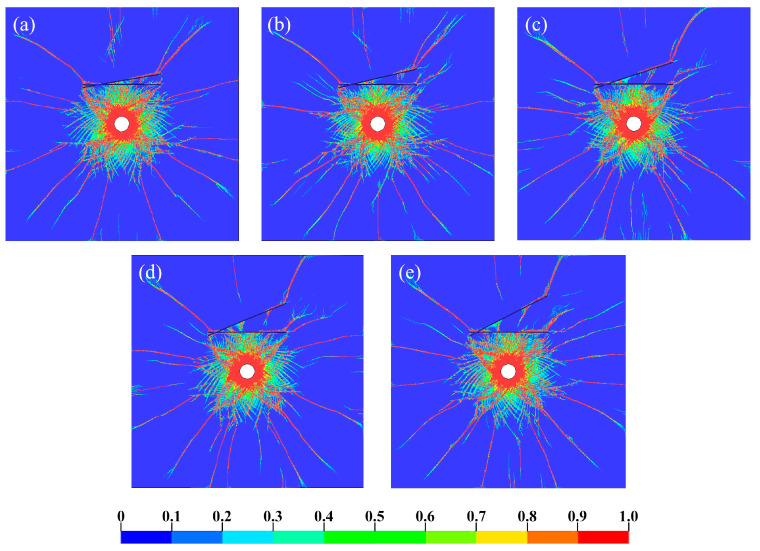
Crack propagation of rock under D = 10 mm with (**a**) θ = 10°; (**b**) θ = 15°; (**c**) θ = 20°; (**d**) θ = 25°; (**e**) θ = 30°.

When the joint thickness D is 10 mm, how the damage mode of the rock mass under different joint angles appears is shown in Figure 4. Cracks in the crushed zone around the blast hole after the explosion were very dense and coarse, showing a radial shape and spreading evenly to the surroundings. The cracks are dense at joint tips and the intersection of two joints due to the stress concentration. After reflecting off Joint I, the stress wave undergoes a reflection enhancement phenomenon, which results in an increase in the stress on the element in front of the reflection. As a result, there are denser and coarser cracks between the blast hole and Joint I compared to intact rock mass.

By comparison, it can be observed that when the joint is thin, the crack propagation pattern is primarily controlled by the angle of the second joint. This is because a thinner tip creates a more pronounced stress concentration. A larger angle causes the wave to reflect and diffract later at the right end of the second joint, leading to the initiation of wing cracks and through-going failure of the rock bridge at the joint tip.

**Figure 5:**
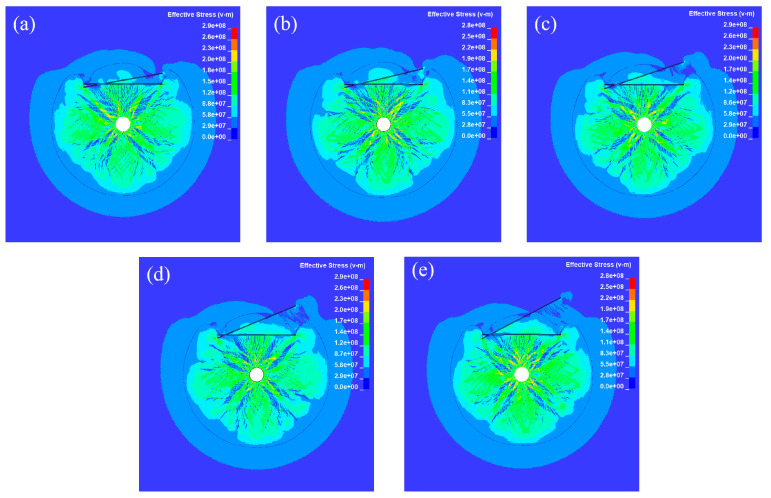
Propagation of the effective stress 0.3 ms after detonating with (**a**) θ = 10°; (**b**) θ = 15°; (**c**) θ = 20°; (**d**) θ = 25°; (**e**) θ = 30°.

Figure 5 shows the stress propagation process corresponding to the conditions in Figure 4, and stress contour maps at 0.3 ms after detonation are selected here. After detonation, viscoelastic joints effectively obstruct the propagation of the stress waves. When the stress wave propagates to Joint I, the reflected wave collides with the incident wavefront. This neutralizes some of the energy, reducing the rock stress between Joint I and the borehole. In addition, when the angle θ is smaller, less energy escapes between joints. When θ is large, the multiple reflections of stress waves between joints are more noticeable (see Figure 4e), with energy primarily transmitted and dissipated between the joints.

The velocity transmission coefficient can be expressed as the ratio of the particle velocities measured on both sides of the joint. When the measured segment includes the entire crossed joint, this transmission coefficient is defined as the overall transmission coefficient of the crossed joints. Therefore, observation points are taken on each side of Joint I and II along the *Y*-axis direction here. By collecting the Y-direction velocities at four observation points, the velocity transmission coefficients of the joints are analyzed to determine the impact of the viscoelastic joints on stress wave propagation.

**Figure 6:**
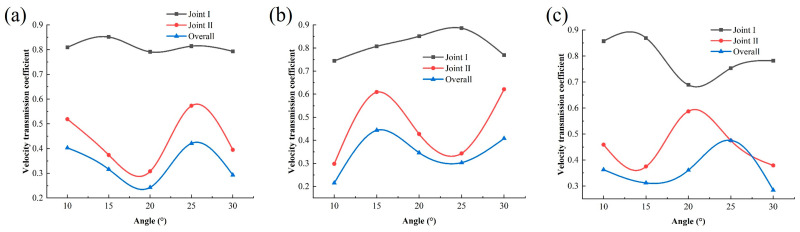
Velocity transmission coefficients of models with different angles: (**a**) 10 mm joints; (**b**) 20 mm joints; (**c**) 30 mm joints.

Figure 6 shows the velocity transmission coefficients of joints with different angles under three conditions of joint thickness. The overall transmission coefficient remains below 40%, and its variation trend with the joint angle is basically similar to that of Joint II. Since the reflection of the stress wave mainly occurs on the incident surface of Joint I, the intensity of the reflected tensile wave of Joint I increases. Under the combination with the incident wave, this results in the velocity transmission coefficient of Joint I being relatively higher compared to Joint II.

Therefore, it can be considered that Joint II plays the main role in weakening the response velocity of the rock mass. It can be understood that Joint I is assumed to be horizontal in the model, meaning that waves propagating in the vertical direction pass through Joint I without significant changes in direction. However, when waves encounter Joint II, which is inclined at different angles, their direction changes significantly, resulting in a more pronounced variation in the y-direction velocity.

When the joint is relatively thin, the stress wave mainly transmits through. When the joint is thicker, it is effectively obstructed, leading to primarily reflection. Additionally, with the varying joint angles and the complexity of multiple stress wave reflections, it is necessary to investigate the impact of joint thickness on stress wave propagation.

### 4.2. The Effect of Joint Thickness D on Stress Wave Propagation

**Figure 7:**
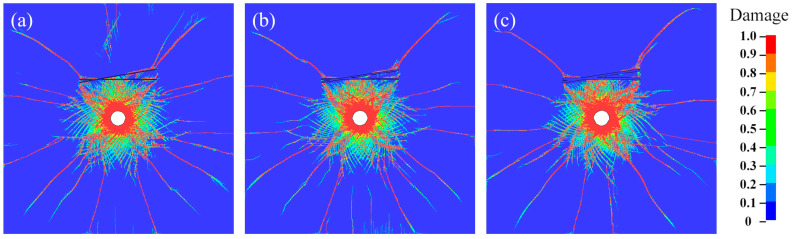
Crack propagation of rock under θ = 20° with (**a**) D = 10 mm; (**b**) D = 20 mm; (**c**) D = 30 mm.

**Figure 8:**
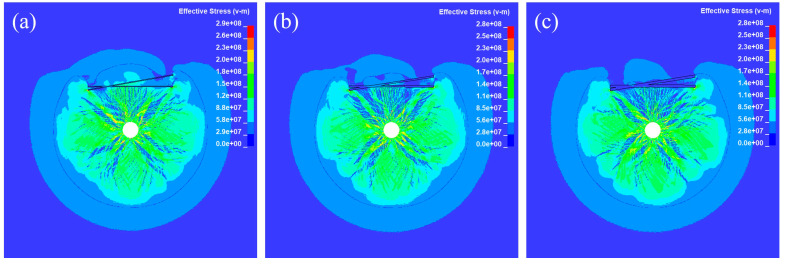
Propagation of the effective stress 0.3 ms after detonating with (**a**) D = 10 mm; (**b**) D = 20 mm; (**c**) D = 30 mm.

Figure 7 and Figure 8 show the crack propagation and the corresponding stress contour maps under different joint thickness D conditions when θ = 10° for double joints, respectively. Increasing the joint thickness effectively obstructs crack propagation and stress wave transmission, and the effect is proportional to the thickness.

**Figure 9:**
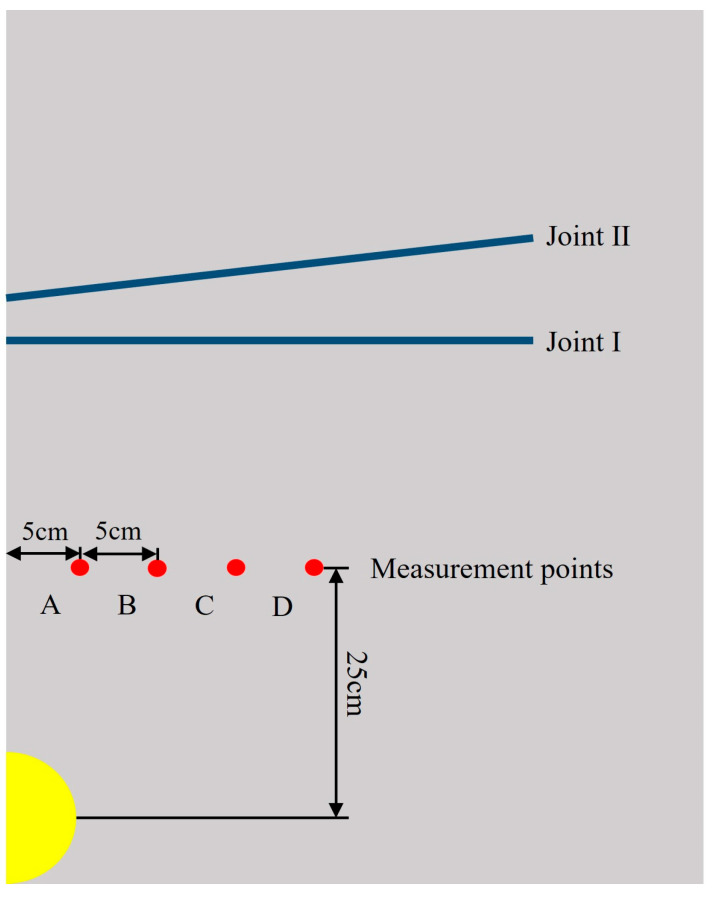
Positions of reference points A, B, C, and D.

**Figure 10:**
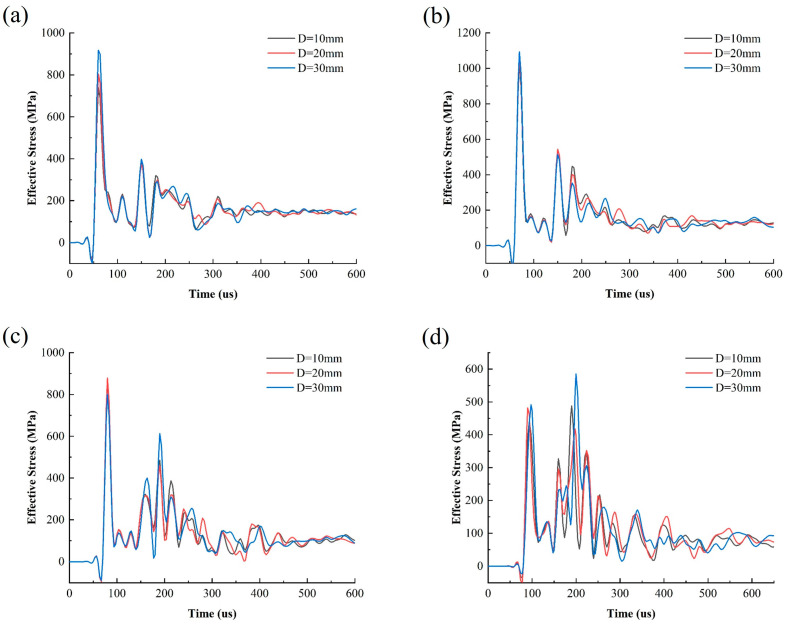
Time–history of effective stress at (**a**) reference A; (**b**) reference B; (**c**) reference C; (**d**) reference D.

To further analyze the impact of joint thickness on crack propagation parallel to the joints, the model with θ = 10 and D = 10 mm is taken as an example. In the middle of Joint I and the borehole, four measurement points, A, B, C, and D, are selected from left to right (see Figure 9). The results from reading the stress data from each point and plotting the effective stress time–history curves under three different joint thicknesses are shown in Figure 10.

It can be seen from Figure 10 that under the same angle and thickness conditions, the peak effective stress gradually decreases with the increase in the burst distance from A to D. It decreases from 350 MPa to around 200 MPa, consistent with classical blasting theory. Meanwhile, as the joint thickness increases, the peak stress of the reflected wave also increases. This indicates that the combination of incident waves and reflected waves promotes the propagation of parallel cracks between the joint and borehole. Moreover, this enhancement effect is proportional to the joint thickness.

**Figure 11:**
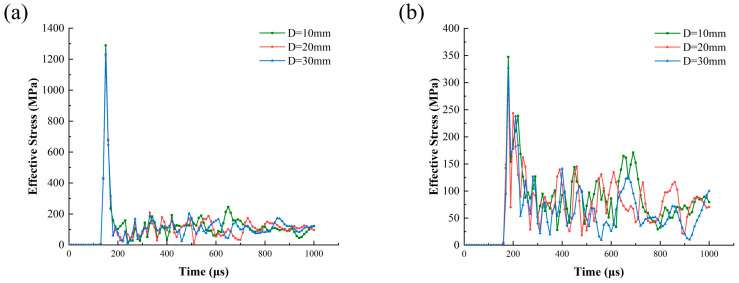
Time–history of effective stress at the ends of different joint thicknesses: (**a**) Joint I; (**b**) Joint II.

To further explore the influence of joint thickness on the crack propagation mechanism at the tip, effective stress data on the outside of the two joint end points were collected. The stress–time–history curves for each point are plotted in Figure 11. At the end of Joint I, the effective stress is relatively large when thinner, reaching around 1300 MPa. The stress at the tip of Joint II is significantly delayed and reduced to around 350 MPa. Due to reflections and diffraction waves between the joints, the stress at the end of Joint II changes irregularly.

By comparing Figure 9 and Figure 10, it can be seen that the peak effective stress at the end of Joint I is significantly higher than that at the same distance from the blast center. In contrast, the stress concentration phenomenon at the end point of Joint II is weaker. It can be seen from Figure 11b that as D increases from 10 mm to 30 mm, the peak effective stress at the joint end decreases from 347 MPa to 327 MPa. This indicates that the increase in D leads to a reduction in stress concentration at the joint end and a decrease in crack propagation.

### 4.3. Extension of the Joints Under Blasting

#### 4.3.1. Distribution of Principal Stresses Around the Tip of the Joints

As discussed in Section 4.1 and Section 4.2, both the angle and thickness of the joints influence not only the crack propagation around the borehole but also the crack behavior at the joint ends. The stress concentration at the joint ends leads to variations in crack propagation patterns under different conditions. Therefore, it is essential to study the stress distribution at the joint ends under explosive loading.

**Figure 12:**
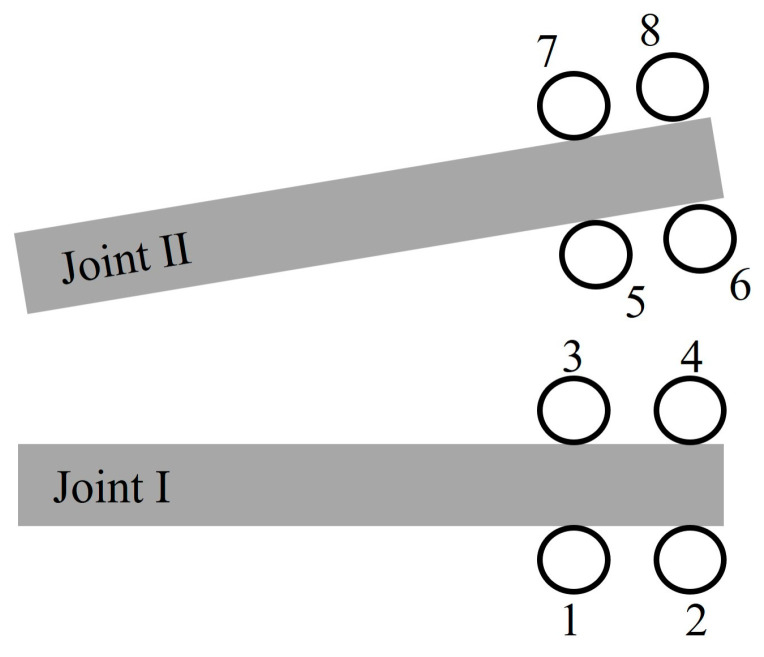
Arrangement of the measurement points (MPs) surrounding the right tips of two joints.

**Figure 13:**
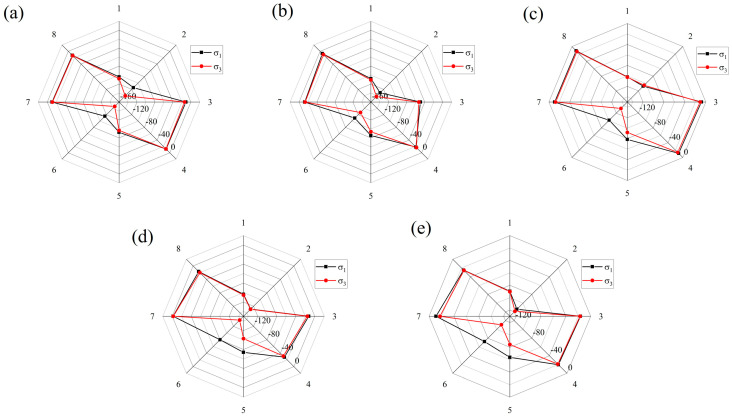
Principal stress distribution surrounding the tip of the D = 20 mm joints 0.4 ms after detonating (MPa): (**a**) θ = 10°; (**b**) θ = 15°; (**c**) θ = 20°; (**d**) θ = 25°; (**e**) θ = 30°.

As shown in Figure 12, eight measurement points are placed around the right end points of two joints with a diameter of 20 mm. The spacing between each point is 1 cm. Figure 13 illustrates the distribution of principal stress at the joint end points with a diameter of 20 mm, observed at 0.4 ms after detonation for different joint angles. Positive values indicate compression. σ_1_ and σ_3_ represent the major and minor principal stresses, respectively.

Generally, tensile stresses dominate around the tips of the joints. On the side of the joint closer to the charge (MP-1, 2, 5, and 6), both σ_1_ and σ_3_ are relatively large. However, due to the obstruction of the joints, the amplitude of the principal stress remains at a relatively low level away from the borehole.

Additionally, the joint angle plays a crucial role in determining the stress distribution at the tips of the joints. The stress difference (σ_1_ − σ_3_) is a key factor affecting rock failure, with an increase observed as the joint angle θ increases. At measurement points MP-5 and MP-6, the principal stress differences are 4.3 MPa and 30.7 MPa at a joint angle of 10° and 20.6 MPa and 38.2 MPa at 30°, respectively. These findings are consistent with the previous simulation, where cracks at the joint ends were densely distributed and connected to cracks in the crush zone.

#### 4.3.2. Displacements Around the Tip of Joints

**Figure 14:**
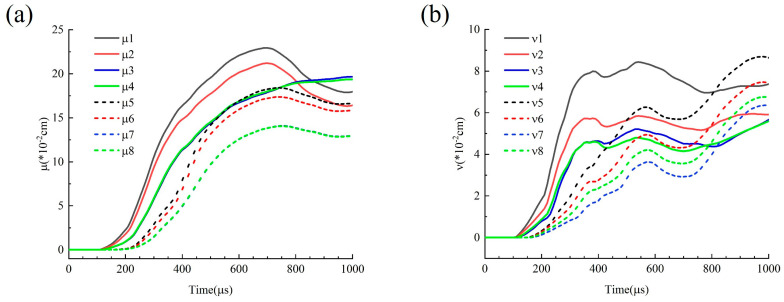
Displacements around the tip of the joints under D = 10 mm, θ = 20° conditions: (**a**) shear displacement μ; (**b**) normal displacement υ.

Figure 14 shows the displacement around the right tip of the 20° joint model at a thickness of 10 mm, with the measurement points arranged in the same configuration as those in Figure 12. In terms of tangential displacement, MP-1, 2, 5, 6 on the explosion-facing side of the joint gradually moves in the positive direction, and as the distance from the explosion center increases, the displacement peak becomes smaller. This means that as the distance from the explosion center increases, the shear deformation decreases. On the back side, the tangential displacement curves at MP-3, 4, 7, 8 tend to overlap, indicating that the right tip of the joint is subjected to tangential compression. The changes in normal displacement exhibit a trend analogous to those observed in tangential displacement. The displacement changes at the tip of Joint I are more drastic than those of Joint II, and there is an attenuation effect as the distance increases. Due to the influence of reflection and transmission, the normal displacement at the measurement points shows more pronounced fluctuations compared to the tangential displacement.

## 5. Conclusions

Based on stress wave fluctuation theory and finite element models, this study presents a thorough investigation into the effects of multiple viscoelastic joints on the blasting behavior in rock masses. The RHT model and Poynting–Thomson model were used to describe the rock mass and joint fillings. Firstly, this study analyzes the impact of joint angle and thickness on the evolution of rock damage. Simultaneously, according to the stress and displacement around the joints, the deformation characteristics of the joints are further elucidated. From the numerical results, the following key conclusions are derived:

(1) The presence of intersecting viscoelastic joints in the rock mass tends to cause a stress concentration, with the intensity inversely proportional to the joint thickness D. At the same time, viscoelastic joints significantly attenuate and obstruct the propagation of blast stress waves. The extent of attenuation grows with the number of joints.
(2) Under explosive loading, the initial cracks at the intersections of crossed joints in the rock mass are all wing cracks. The larger the joint filling thickness D, the better the blasting effect on the rock mass at the blast-facing side of Joint I, and the worse the effect on the back side. The extension of wing cracks at Joint II is primarily determined by θ. As θ increases, the cracks on the back side of the joint become fewer and thinner, and the rock failure mode shifts from tensile failure to shear-tensile failure. Owing to the reflected waves in front of Joint I, the dense cracks in the crush zone surrounding the borehole extend to the joint surface, creating longitudinal cracks in the joint and connecting with the transverse cracks, resulting in improved fragmentation in that area.
(3) The larger θ and D, the more pronounced the effect of the joints on wave speed. When the joint is thin, stress waves are predominantly transmitted. When the joint is thick, reflection becomes the primary mode of wave interaction. Among them, Joint II primarily plays a role in reducing the stress wave velocity, accounting for an average of 68.9% of the overall weakening effect. The difference in principal stress on the blast-facing side of the joint increases with θ, showing increases of 7.5 MPa and 16.3 MPa, which is strongly correlated with the rock mass damage.

In this study, we focused on investigating the effects of viscoelastic joints on rock masses by separately considering the influence of joint thickness and angle. However, more complex scenarios, such as the combined effects of thickness and angle, random joint distributions, and various joint filling materials, remain to be explored. These aspects will be addressed in future studies to further enhance our understanding of the dynamic behavior of jointed rock masses under blasting loads.

## Figures and Tables

**Table 1:** Model parameters of explosive.

ρ (kg·m^−3^)	D (m·s^−1^)	P_CJ_ (GPa)	A (GPa)	B (GPa)	R_1_	R_2_	ω	E_0_ (GPa)
1320	6690	16	586	21.6	5.81	1.77	0.282	7.38

**Table 2:** Model parameters of rock.

ρ (kg·m^−3^)	G (GPa)	$f_{c}$ (MPa)	$p_{el}$ (MPa)	$p_{co}$ (GPa)
2700	24.17	119	40	6

**Table 3:** Model parameters of air.

ρ (kg·m^−3^)	C4	C5	E_0_ (GPa)
1.29	0.4	0.4	0.025

**Table 4:** Model parameters of joints.

ρ (kg·m^−3^)	E (GPa)	$G_{0}$ (GPa)	$G_{L}$ (GPa)	β
1160	0.585	0.591	0.126	7 × 10^−4^

## Data Availability

The original contributions presented in this study are included in the article. Further inquiries can be directed to the corresponding author.

## Abbreviations

The following abbreviations are used in this manuscript:

TDRM	time-domain recursive method
SPH	smoothed particle hydrodynamics
FEM	finite element method
DEM	discrete element method
DSIF	dynamic stress intensity factor
DIC	digital image correlation
RHT	Riedel–Hiermaier–Thoma
JWL	Jones-Wilkins-Lee
